# A new method to estimate parameters of the growth model for metastatic tumours

**DOI:** 10.1186/1742-4682-10-31

**Published:** 2013-05-09

**Authors:** Esmaeil Mehrara, Eva Forssell-Aronsson, Viktor Johanson, Lars Kölby, Ragnar Hultborn, Peter Bernhardt

**Affiliations:** 1 Department of Radiation Physics, University of Gothenburg, Sahlgrenska University Hospital, Göteborg, SE - 413 45, Sweden; 2 Department of Surgery, University of Gothenburg, Göteborg, Sweden; 3 Department of Oncology, University of Gothenburg, Göteborg, Sweden; 4 Department of Medical physics and Biomedical Engineering, Sahlgrenska University Hospital, Göteborg, Sweden

**Keywords:** Modelling tumour growth, Metastasis, Dissemination, Gompertzian

## Abstract

**Purpose:**

Knowledge of natural tumour growth is valuable for understanding tumour biology, optimising screening programs, prognostication, optimal scheduling of chemotherapy, and assessing tumour spread. However, mathematical modelling in individuals is hampered by the limited data available. We aimed to develop a method to estimate parameters of the growth model and formation rate of metastases in individual patients.

**Materials and methods:**

Data from one patient with liver metastases from a primary ileum carcinoid and one patient with lung metastases from a primary renal cell carcinoma were used to demonstrate this new method. Metastatic growth models were estimated by direct curve fitting, as well as with the new proposed method based on the relationship between tumour growth rate and tumour volume. The new model was derived from the Gompertzian growth model by eliminating the time factor (age of metastases), which made it possible to perform the calculations using data from all metastases in each patient. Finally, the formation time of each metastasis and, consecutively, the formation rate of metastases in each patient were estimated.

**Results:**

With limited measurements in clinical studies, fitting different growth curves was insufficient to estimate true tumour growth, even if patients were followed for several years. Growth of liver metastases was well described with a general growth model for all metastases. However, the lung metastases from renal cell carcinoma were better described by heterogeneous exponential growth with various growth rates.

**Conclusion:**

Analysis of the regression of tumour growth rate with the logarithm of tumour volume can be used to estimate parameters of the tumour growth model and metastasis formation rates, and therefore the number and size distribution of metastases in individuals.

## Introduction

Studying natural tumour growth is valuable for understanding tumour biology, optimising screening programs, prognostication [1], optimal scheduling of chemotherapy [2], and assessing tumour spread (number and size distribution of metastases, including micro-metastases) [3,4]. Mathematical models such as the exponential and the Gompertzian growth models are usually used to describe tumour growth. The growth of a tumour can be described by a curve defined by the selected model, where the tumour volume (V) is a function of time (t): V = f(t).

The exponential model is a simple growth model of solid tumours. Assuming exponential tumour growth, the growth rate can be calculated from tumour volume measurements from at least two occasions, either as the tumour volume doubling time (DT) or the specific growth rate (SGR) [5]. DT is the time needed for a tumour to double its volume, and SGR is the relative volume increase per unit time, given in %/day. If the tumour volume is measured at times t_0_ and t, the following equation is valid [5]:

(1) $$V=V_{0}e^{\mathrm{SGR}\left(t-t_{0}\right)}$$

The above equation shows that SGR is equivalent to the exponential growth constant of the tumour. In other words:

(2) $$\mathrm{SGR}=\frac{1}{V}\frac{\mathrm{dV}}{\mathrm{dt}}$$

If tumour volume is measured on two occasions, t_1_ and t_2_, then the SGR of the tumour during the period of observation can be calculated as:

(3) $$\mathrm{SGR}=\frac{\ln\left(V_{2}/V_{1}\right)}{t_{2}-t_{1}}$$

where V_1_ and V_2_ are the tumour volume at t = t_1_ and t_2_, respectively. SGR is reciprocally related to DT as follows:

(4) $$\mathrm{SGR}=\frac{\ln\left(2\right)}{\mathrm{DT}}.$$

We have previously demonstrated that SGR is less affected by measurement uncertainties than DT, and is a better variable to characterise tumour growth rate [5,6].

According to the exponential model, the tumour’s SGR is constant and independent of tumour volume. However, studies have shown that tumour growth rate may decline with time [7-9] (i.e., non-exponential growth).

The Gompertzian model is widely used to describe the growth of non-exponentially growing tumours. According to the Gompertzian growth model, the variation of tumour volume with respect to time is as follows:

(5) $$V=V_{0}e^{\frac{{\mathrm{SGR}}_{0}}{\lambda }\left(1-e^{-\lambda \left(t-t_{0}\right)}\right)}$$

where SGR_0_ is the initial tumour growth rate at t = t_0_, and λ is the growth deceleration constant. When λ approaches zero, the Equation 5 reduces to Equation 1 (i.e., the exponential growth model).

The standard method to find the growth model that best describes tumour growth is direct curve fitting. Exponential and Gompertzian growth curves are fitted to the volume of each tumour, and the model with the best fit is selected. Using direct curve fitting with the Gompertzian model requires at least three data points per tumour. However, in clinical studies, therapy is usually initiated soon after diagnosis; therefore, the natural tumour growth patterns can only be followed for a limited time before the onset of treatment. Thus, clinical observations of growth rate decline in tumours (i.e., non-exponential growth curves) are very rare, and the exponential model is most commonly used [10].

The aim of this study was to develop a new method to estimate the parameters of the non-exponential growth model for metastatic tumours in patients. To demonstrate the applicability of the new proposed method, we applied the method to data from one patient with liver metastases arising from a primary ileum carcinoid and one patient with lung metastases arising from a primary renal cell carcinoma.

## Materials and methods

### Patients

Data from two patients were used in this study. The first patient was diagnosed with primary midgut carcinoid and liver metastases. The primary tumour was surgically resected in 1995. Growth data were obtained from eight computed tomography (CT) examinations performed annually during 1995–2002. During this period, the patient was treated with octreotide (Sandostatin, Sandoz/Novartis, Basel, Switzerland) for hormonal symptom relief, and interferon alfa-2b (IntronA, Schering-Plough Corporation, New Jersey, USA) was administered on three occasions without clinical response. The volume of each tumour was measured by point counting: a transparent paper marked with square millimeters was used to measure the tumour area in CT slices, and the tumour volume in the slice was estimated by multiplying the tumour area by the slice thickness. The total tumour volume was calculated as the sum of tumour volumes from the individual CT slices.

The second patient was diagnosed with primary renal cell carcinoma with lung metastases. Renal cancer is notoriously chemotherapy resistant. During the 1980th biological therapy with interferon with few responses, but considerable toxicity, was introduced and in the 1990th also IL2 was added, again with modest efficacy but with significant side effects [11]. Due to the frequently very slow disease progression with few symptoms (as in this case), many oncologists in the Scandinavian countries preferred expectation. The frequent X-ray investigations were probably done for psychological reasons. Therefore, this patient was untreated and we studied the natural growth of seven lung metastases in this patient. A total of 32 conventional two-dimensional AP chest radiographs collected from 1989 to 1999 were available. The area of each tumour in each radiograph was estimated using Osirix (cf. http://www.osirix-viewer.com/). Each tumour was assumed to be equal to the volume of a sphere with the diameter of a circle with the same area as the estimated tumour area in the radiograph. Because the lining border of the tumour could not be clearly defined in all images, the number of available data points may vary for different metastatic tumour masses.

All CT and X-ray imaging data used in this study concern deceased persons and were retrieved retrospectively from the patient imaging database at Sahlgrenska University Hospital, Gothenburg, Sweden. This type of information on deceased persons is exempt from ethical approval according to the Ethical Review Act in Sweden (2003:460).

### Studying the tumour growth model

We first attempted to estimate the growth model of each tumour by direct curve fitting, where it was not possible to select the most probable growth model of each metastasis directly. However, exponential growth rates were higher for smaller tumours than for larger tumours, possibly as a result of growth deceleration as tumours grow (the Gompertzian growth model). Therefore, we developed a new mathematical method assuming that, in each patient, the smaller metastases represent the growth of larger metastases when they were of small size and vice versa. Based on this assumption, all metastases of the same type, in the same tissue and in the same patient, follow a general Gompertzian growth model; variations in growth rates are because of volumetric differences. If the time of formation of each metastasis were available, it would be possible to estimate the parameters of such a general Gompertzian model by fitting the Gompertzian curve to data from all metastases in a single curve fitting. However, the formation time of each metastasis can, in turn, be estimated only if the growth model of the metastasis is known. Therefore, we reformulated the Gompertzian curve (i.e., Equation 5) by eliminating the time parameter. From Equations 2 and 5, the relation between SGR and tumour volume is as follows (see Appendix A):

(6) $$\mathrm{SGR}=\mathrm{SG}R_{0}-\lambda \;\ln\left(V/V_{0}\right)$$

Equation 6 does not include time, which makes it possible to use data from all metastases of the same type in a single patient without knowing the age of each individual tumour. Equation 6 shows that the regression of tumour SGR with the logarithm of its volume is linear if the growth model is Gompertzian. Therefore, the parameters of the general Gompertzian growth model of metastasis in each patient (SGR_0_ and λ) can be estimated using the linear regression parameters in Equation 6.

This method was applied to our patient data as follows. (1) SGR values were calculated for each metastasis, using Equation 3 for each pair of consecutive tumour volume measurements. (2) The logarithm of the geometric mean of the two volumes (used in stage 1) was calculated for each pair of consecutive tumour volume measurements of each metastasis. (3) Using all SGRs (stage 1) and volumes (stage 2) from all metastases in each patient, λ and SGR_0_ were estimated using the linear regression parameters in Equation 6, assuming V_0_ = 10^-9^ cm^3^ (one cell). (4) Equation 5 with the estimated λ and SGR_0_ values (from stage 3) was assumed to represent the general Gompertzian growth curve of all metastases in each patient. According to this assumption, the general growth curve can describe the growth of each metastasis when the time origin is changed (i.e., the curve is shifted backward and forward). Therefore, (5) the general growth curve was fitted to the volume of each metastasis with time origin as a variable, and the formation time of each metastasis was estimated using the best fit for each tumour.

All curve fittings were performed using Matlab 6.5.1 with the curve fitting toolbox (The MathWorks, USA).

## Results

**Figure 1:**
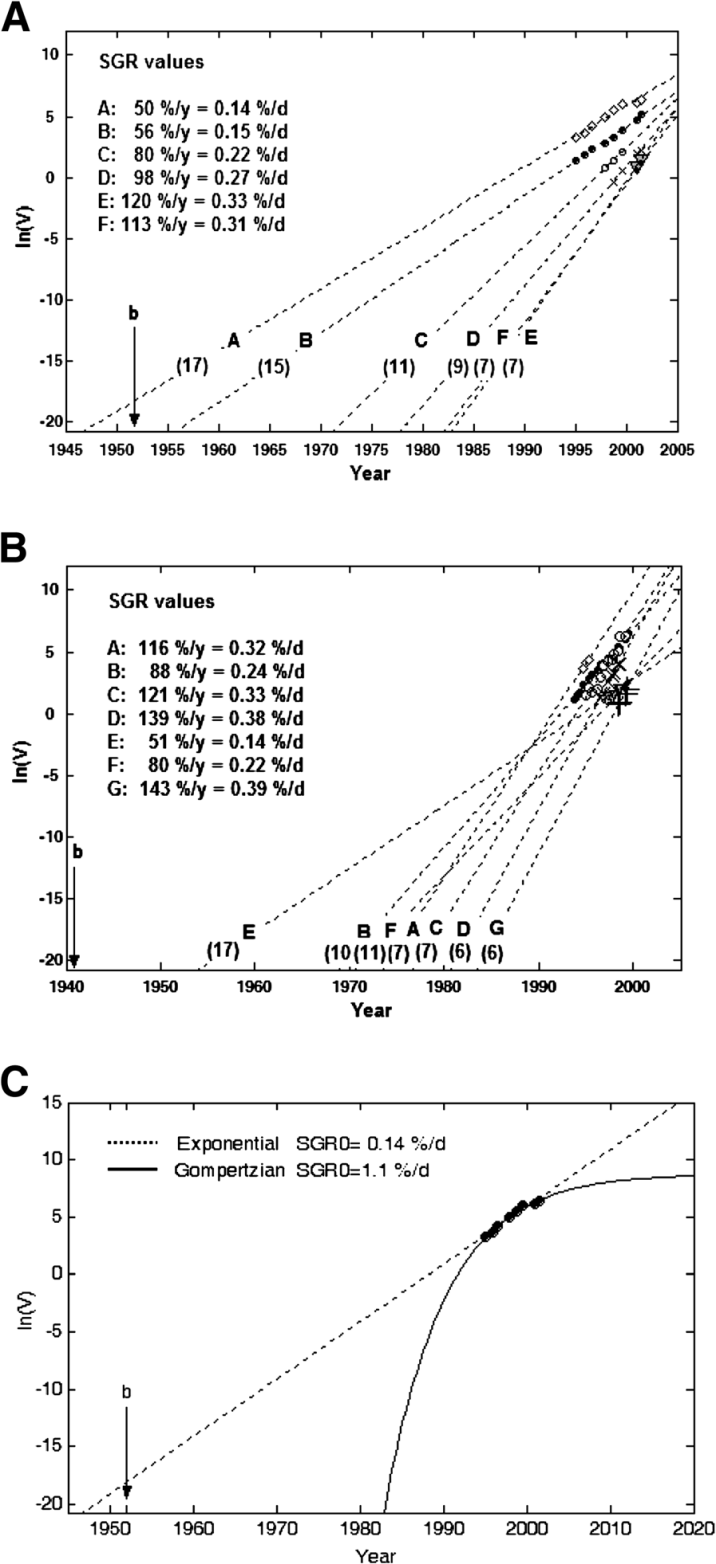
**The logarithm of tumour volume vs. time for all metastases in the liver (A) and lungs (B), with corresponding exponential growth fit to each metastasis.** SGR is given for each tumour; the values in parentheses for each line depict the doubling time in months. A trend of decreasing growth rate (slope of line) from large to small tumours is visible for liver metastases, but not for lung metastases. **C** The best exponential (dashed line) and Gompertzian (solid line) model curve fits to the logarithm of the volume of liver metastasis A with extrapolation to the volume of one cell. b represents the birth of the patient.

For both patients, it was possible to examine direct curve fitting for most tumours because the tumours had been followed for relatively long periods of time. The volume of each tumour, in the liver (except metastases E and F) or in the lungs, was well described by either the exponential or the Gompertzian model with high r^2^ values (Table 1). Liver metastases E and F were only observed on two occasions, and the Gompertzian model requires three data points for curve fitting. Based on the results of the direct model fitting, it was not possible to select the most probable growth model for each tumour. However, the estimated tumour formation times and SGR_0_ values differed when estimated by the different models. The estimated formation year of liver tumour A, obtained by the exponential fit model, was 1947, which is 5 years before the birth of the patient (1952) and, therefore, not realistic. For the best exponential fits, the SGR values were 0.14-0.33%/day for liver metastases (Figure 1A) and 0.14-0.39%/day for lung metastasis (Figure 1B), respectively. These values correspond to DT values of 7–17 and 6–17 months, respectively.

**Table 1:** Results of direct curve fitting of the exponential and the Gompertzian growth models to tumour volume data from two patients

**Patient (year of birth)**	**Tumour type**	**Tumour (V cm**^**3**^**)**	**Number of data points**	**Growth model**						
				**Exponential**			**Gompertzian**			
				**SGR**_**0**_**(%/day)**	**Year of formation**	**r**^**2**^	**SGR**_**0**_**(%/day)**	**λ (1/d)**	**Year of formation**	**r**^**2**^
1 (1952)	Liver metastases from a primary midgut carcinoid	A (614)	8	0.14	1947	0.972	1.1	0.0004	1983	0.988
		B (171)	8	0.15	1956	0.992	0.2	0	1956	0.989
		C (8)	3	0.22	1971	1.000	0.3	0	1976	0.954
		D (9)	4	0.27	1978	0.997	1.3	0.0005	1991	1.000
		E (4)	2	0.33	1982	1.000	-	-	-	-
		F (3)	2	0.31	1982	1.000	-	-	-	-
2 (1941)	Lung metastases from a primary renal cell carcinoma	A (82)	3	0.32	1973	0.998	3.8	0.0014	1992	1.000
		B (635)	19	0.24	1968	0.992	0.5	0.0001	1977	0.993
		C (489)	12	0.33	1976	0.939	0.4	0	1977	0.938
		D (54)	7	0.38	1980	0.986	1.8	0.0006	1991	0.990
		E (8)	6	0.14	1953	0.798	0.1	0	1953	0.791
		F (11)	5	0.22	1970	0.946	0.4	0.0001	1978	0.944
		G (7)	4	0.39	1983	0.998	0.5	0	1987	0.970

SGR_0_, r, and λ are the SGR value at the time of tumour formation, the correlation coefficient, and the Gompertzian growth deceleration constant, respectively. Curve fitting of the Gompertzian model was not possible for liver tumours E and F because too few data points were available. V: Maximum tumour volume.

Figure 1C shows the best exponential and Gompertzian model curve fits for the volume of liver metastasis A. Although both models fit well with tumour volume in a short time interval, the extrapolated tumour formation times differed by a large margin: 1947 and 1982 with the exponential and Gompertzian models, respectively. The estimated SGR at time of tumour formation (SGR_0_) differed by an order of magnitude: 0.14%/day and 1.1%/day with the exponential and the Gompertzian models, respectively. These values correspond to DT values of 17 months and 2 months, respectively.

**Figure 2:**
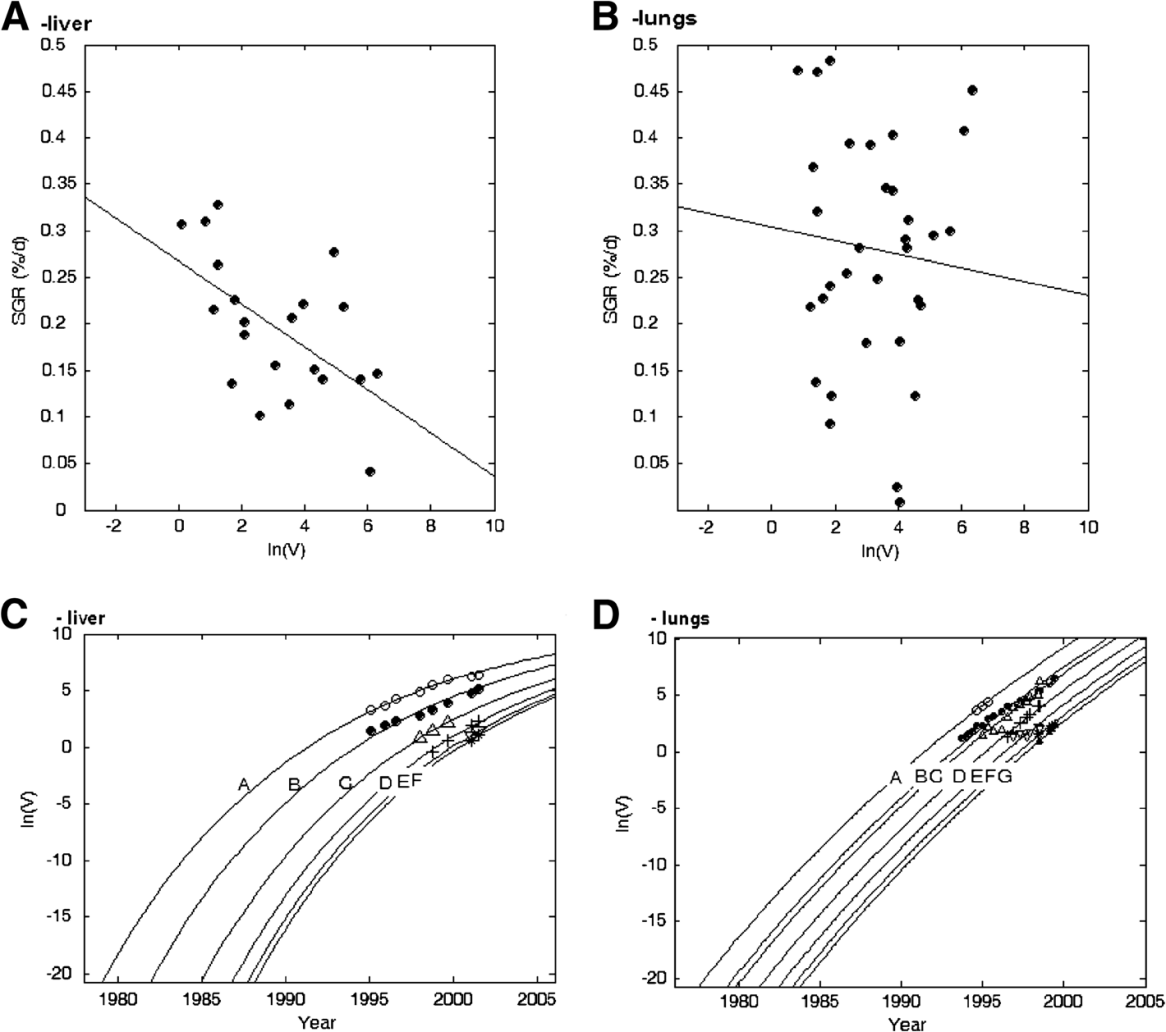
**SGR vs. the logarithm of the volume of the metastases in the liver (A) and the lungs (B).** The best linear regression fits are shown. The correlation was statistically significant in the liver (r^2^ = 0.33, *p* < 0.005), but not in the lungs. The logarithm of the tumour volume vs. time for all metastases in the liver (**C**) and the lungs (**D**) with the general Gompertzian growth model curve fits.

For the liver metastases, the negative correlation between SGR and the logarithm of tumour volume was statistically significant (r^2^ = 0.33, *p* < 0.005), and the estimated λ and SGR_0_ values were 0.00023 and 0.79%/day, respectively (Figure 2A). For the lung metastases, the correlation was not statistically significant. However, the estimated λ and SGR_0_ values were 0.00007 and 0.46%/day, respectively (Figure 2B). Curve fitting of the general Gompertzian growth model to data for the metastases in each patient are depicted in Figure 2C,D. In each patient, the same growth curve was shifted in time to fit the volume of each metastasis.

**Figure 3:**
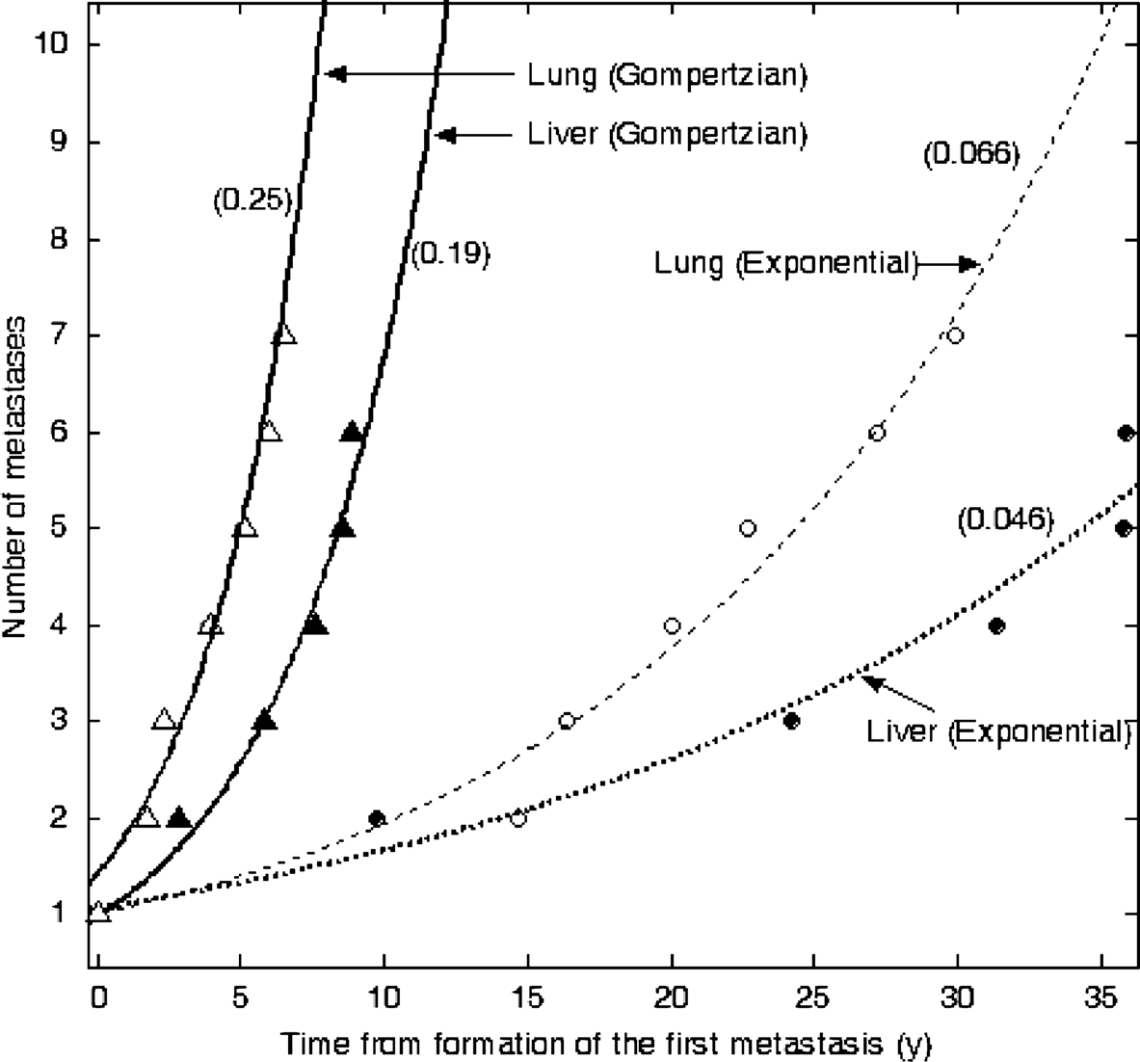
**The number of metastases vs. the time from formation of the first metastasis.** Metastasis formation rates were determined for liver and lung metastases according to the exponential and Gompertzian growth models. Values in parentheses represent the constant of the exponential increase rate (per year).

Figure 3 depicts the number of metastases as a function of time in each patient. The number of metastases increased exponentially with respect to time, assuming that the tumours grow exponentially with different growth rates or according to a general Gompertzian model. The increase rate of the number of metastases based on the general Gompertzian model was higher than the rate based on the exponential model.

## Discussion

Our results demonstrate that, when the observation of tumour growth is limited in time, fitting of different growth curves to the volume of each tumour is not sufficient to estimate the true metastatic growth. In two patients, direct curve fitting was insufficient even when metastatic growths were followed for several years. Selection of the correct tumour growth model is crucial for further analyses of metastatic formation rate, number of metastases present, and response estimates in targeted radionuclide therapy [8].

Because the data available in clinical studies is limited, the exponential model is often used to characterise tumour growth. However, in the present study we demonstrated that extrapolation of different growth curves can generate diverse tumour formation times and metastasis formation rates (Figure 1C). This problem has also been addressed before, and some attempts to handle limited data more efficiently have been proposed. One method to assess tumour growth decline in clinical studies has been to calculate the correlation between DT and tumour volume [12,13]. However, by definition this technique is not mathematically valid according to the Gompertzian growth model. The present study was therefore based on the linear regression of tumour SGR with the logarithm of tumour volume, a relation that was obtained by reformulating the Gompertzian model. In addition, our approach enabled us to estimate metastasis formation times and rates. Akanuma previously attempted to find the model constants for the Gompertzian growth model using the linear correlation between growth rate and the logarithm of tumour volume [14]. Akanuma’s method was based on a graphic estimation of SGR at different tumour volumes. Tumours were scaled according to their doubling time, and very high or negative values were excluded. However, we have previously shown that negative and zero values should not be excluded from such calculations [5,6].

In the proposed method, a significant negative correlation between SGR and the logarithm of tumour volume indicates that growth deceleration is a dominating factor in the observed growth rate variations. In other words, the smaller tumours represent the growth of larger tumours when they were of small size and vice versa, and a general Gompertzian growth model (with specific SGR_0_ and λ values) can describe the growth of all tumours. Lack of correlation between SGR and the logarithm of tumour volume indicates that biological factors other than growth deceleration dominate the observed growth rate variations. Thus, these tumours may grow exponentially with different growth rates or according to the Gompertzian model, but the model constants (SGR_0_ and λ) are heterogeneously distributed among tumours.

According to the linear regression of tumour SGR with the logarithm of tumour volume, the liver metastases in the carcinoid patient probably grow according to a general Gompertzian growth model. This patient was treated with octreotide. Because, based on curve fitting results, no tumours in this patient deviated from exponential growth, the treatment was assumed to have no effect on tumour growth. The growth of lung metastases in the patient with renal cell carcinoma exhibited high variability with our proposed method, indicating that the renal cancer metastases in the lungs likely grew exponentially with different growth rates. To further strengthen the accuracy of the model selection, we extended our methodology by including analysis of the metastatic formation rate.

The exponential model presented to describe the metastasis formation rate does not depend on the origin of a metastasis (e.g., whether it originates from the primary or a metastatic lesion). The model described well the increase of the number of metastases growing exponentially with different growth rates, or growing according to a general Gompertzian model. This finding is similar to the results of a previous study that employed a different approach [3].

Our results showed that a decelerating growth model such as the Gompertzian model implies a higher metastasis formation rate than the exponential model. However, a higher metastasis formation rate does not necessarily mean a larger number of metastases at any time point, and the number of metastases should be calculated for any specific time to compare different models. The time origin in Figure 3 is time of the formation of the first metastasis, which is different for different models. The estimated number of liver metastases at the time of primary surgery in the ileum carcinoid patient was 9 using the exponential model and 22 using the Gompertzian model. The value of 9 metastases did not reflect reality; 24 metastases were imaged aside from the studied metastases. Therefore, the Gompertzian model provided the best estimate of the number of liver metastases. The estimated number of lung metastases at the time of primary surgery in the renal cell carcinoma patient was 14 according to the exponential model and 84 according to the Gompertzian model. The value of 84 metastases is unlikely to reflect reality, because only one small, non-growing metastasis was imaged aside from the seven lesions studied. If other metastases were present, they should have grown to visible size during 10 years of follow-up. Therefore, the exponential model provided the best estimate of the number of lung metastases. Our results emphasise the importance of having correct tumour growth information to correctly estimate the number and size distribution of metastases.

We evaluated the Gompertzian growth model in the present study because it is the most commonly adopted model in clinical studies [7,8]; however, our approach is theoretically applicable to all growth models.

## Conclusion

Analysis of the regression of tumour growth rate with its volume can be used to estimate the non-exponential growth model parameters of metastatic tumours. These results are valuable for the optimisation of targeted radionuclide therapy based on the estimated number and size distribution of metastases in individual patients.

## Appendix A

Relation between tumour growth rate and tumour volume

From Equation 5 (Gompertzian model):

(7) $$\ln\left(\frac{V}{V_{0}}\right)=\frac{{\mathrm{SGR}}_{0}}{\lambda }\left({1-e}^{-\mathrm{\lambda t}}\right)$$

and

$$\frac{1}{V}\frac{\mathrm{dV}}{\mathrm{dt}}=\frac{{\mathrm{SGR}}_{0}}{\lambda }\left(+,\lambda \right)e^{-\mathrm{\lambda t}}$$

Replacing the left side of the above equation with Equation 2 gives:

$$\mathrm{SGR}={\mathrm{SGR}}_{0}e^{-\mathrm{\lambda t}}$$

or

$$e^{-\mathrm{\lambda t}}=\frac{\mathrm{SGR}}{{\mathrm{SGR}}_{0}}$$

If exp (−λt) in Equation 7 is replaced by the above equation, then:

$$\ln\left(\frac{V}{V_{0}}\right)=\frac{{\mathrm{SGR}}_{0}}{\lambda }\left(1-\frac{\mathrm{SGR}}{{\mathrm{SGR}}_{0}}\right)$$

Readjustment of the above equation gives Equation 6 in the article:

$$\mathrm{SGR}=\mathrm{SG}R_{0}-\lambda \;\ln\left(V/V_{0}\right)$$

where SGR is the value of tumour SGR at each period of observation (calculated using Equation 3 in the article) and V is the geometric mean of tumour volume at that period of observation.

**Figure 4:**
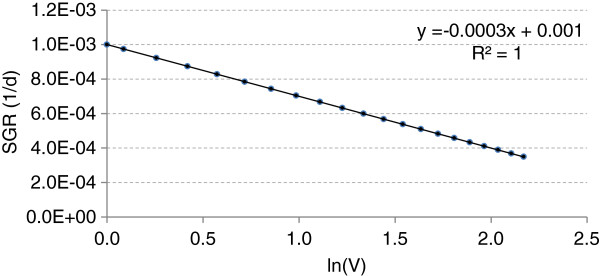
Variation of specific growth rate (SGR) with logarithm of tumour volume.

In the following example, Table 2 and Figure 4, we assume that the SGR_0_ and volume of a tumour at time t = 0 are 0.1%/day (= 0.001 day^-1^) and V_0_ = 1 (arbitrary unit), respectively. The tumour grows according to the Gompertzian model with λ = 0.0003 day^-1^. Tumour volume is measured every six months (180 days). The last two columns in Table 2 show the calculated logarithm of the geometric mean of tumour volume and the tumour SGR at each consecutive pair of measurements, respectively:

**Table 2:** Variation of specific growth rate (SGR) with logarithm of tumour volume

**t (days)**	**t (months)**	**V (Gomp)**	**V (average)**	**ln(V)**	**SGR (day**^**-1**^**)**
0	0	1.00	1.00	0.00	0.00100
180	6	1.19	1.09	0.09	0.00097
360	12	1.41	1.29	0.26	0.00092
540	18	1.65	1.52	0.42	0.00087
720	24	1.91	1.77	0.57	0.00083
900	30	2.20	2.05	0.72	0.00078
1080	36	2.52	2.35	0.86	0.00074
1260	42	2.86	2.68	0.99	0.00070
1440	48	3.22	3.03	1.11	0.00067
1620	54	3.61	3.41	1.23	0.00063
1800	60	4.02	3.81	1.34	0.00060
1980	66	4.45	4.23	1.44	0.00057
2160	72	4.90	4.67	1.54	0.00054
2340	78	5.37	5.13	1.64	0.00051
2520	84	5.86	5.61	1.72	0.00048
2700	90	6.36	6.11	1.81	0.00046
2880	96	6.88	6.62	1.89	0.00043
3060	102	7.41	7.14	1.97	0.00041
3240	108	7.94	7.67	2.04	0.00039
3420	114	8.49	8.21	2.11	0.00037
3600	120	9.04	8.76	2.17	0.00035

Figure 4 shows variation of SGR with the logarithm of tumour volume according to the Table 2. The estimated SGR_0_ and λ values are equal to the assumptions (i.e., 0.001 day^-1^ and 0.0003 day^-1^, respectively).

## Competing interests

The authors declare that they have no competing interests.

## Authors’ contributions

EM and PB initiated the study. EM developed the model and analyzed the data. EM, EFA, PB and RH drafted the manuscript. RH, VJ and LK participated in selecting images and delineating tumorus. All authors read and approved the final manuscript.

## References

[B1] Bassukas ID Hofmockel G Tsatalpas P Eberle V Maurer-Schultze B Prognostic relevance of the intrinsic growth deceleration of the first passage xenografts of human renal cell carcinomas Cancer 1996 78 2170 2172 10.1002/(SICI)1097-0142(19961115)78:10<2170::AID-CNCR19>3.0.CO;2-W 8918411

[B2] Norton L A Gompertzian model of human breast cancer growth Cancer Res 1988 48 7067 7071 3191483

[B3] Withers HR Lee SP Modeling growth kinetics and statistical distribution of oligometastases Semin Radiat Oncol 2006 16 111 119 10.1016/j.semradonc.2005.12.006 16564446

[B4] Iwata K Kawasaki K Shigesada N A dynamical model for the growth and size distribution of multiple metastatic tumors J Theor Biol 2000 203 177 186 10.1006/jtbi.2000.1075 10704301

[B5] Mehrara E Forssell-Aronsson E Ahlman H Bernhardt P Specific growth rate versus doubling time for quantitative characterization of tumor growth rate Cancer Res 2007 67 3970 3975 10.1158/0008-5472.CAN-06-3822 17440113

[B6] Mehrara E Forssell-Aronsson E Ahlman H Bernhardt P Quantitative analysis of tumor growth rate and changes in tumor marker level: specific growth rate versus doubling time Acta Oncol 2009 48 591 597 10.1080/02841860802616736 19330565

[B7] Afenya EK Calderon CP Diverse ideas on the growth kinetics of disseminated cancer cells Bull Math Biol 2000 62 527 542 10.1006/bulm.1999.0165 10812720

[B8] Bajzer Z Gompertzian growth as a self-similar and allometric process Growth Dev Aging 1999 63 3 11 10885854

[B9] Hart D Shochat E Agur Z The growth law of primary breast cancer as inferred from mammography screening trials data Br J Cancer 1998 78 382 387 10.1038/bjc.1998.503 9703287 PMC2063020

[B10] Haeno H Gonen M Davis MB Herman JM Iacobuzio-Donahue CA Michor F Computational modeling of pancreatic cancer reveals kinetics of metastasis suggesting optimum treatment strategies Cell 2012 148 362 375 10.1016/j.cell.2011.11.060 22265421 PMC3289413

[B11] Goey SH Verweij J Stoter G Immunotherapy of metastatic renal cell cancer Ann Oncol 1996 7 887 900 10.1093/oxfordjournals.annonc.a010790 9006738

[B12] Nakamura M Roser F Michel J Jacobs C Samii M The natural history of incidental meningiomas Neurosurgery 2003 53 62 70 discussion 70–61 10.1227/01.NEU.0000068730.76856.58 12823874

[B13] Ozono S Miyao N Igarashi T Marumo K Nakazawa H Fukuda M Tsushima T Tokuda N Kawamura J Murai M Tumor doubling time of renal cell carcinoma measured by CT: collaboration of Japanese society of renal cancer Jpn J Clin Oncol 2004 34 82 85 10.1093/jjco/hyh011 15067101

[B14] Akanuma A Parameter analysis of Gompertzian function growth model in clinical tumors Eur J Cancer 1978 14 681 688 658092 10.1016/0014-2964(78)90304-3

